# Deep whole-genome resequencing sheds light on the distribution and effect of amphioxus SNPs

**DOI:** 10.1186/s12863-022-01038-w

**Published:** 2022-04-08

**Authors:** Yunchi Zhu, Na Lu, J.-Y. Chen, Chunpeng He, Zhen Huang, Zuhong Lu

**Affiliations:** 1 State Key Laboratory of Bioelectronics, Southeast University, Nanjing, Jiangsu China; 2 Nanjing Institute of Paleontology and Geology, Nanjing, China; 3 The Public Service Platform for Industrialization Development Technology of Marine Biological Medicine and Product of State Oceanic Administration, College of Life Sciences, Fujian Normal University, Fuzhou, Fujian China; 4 Key Laboratory of Special Marine Bio-Resources Sustainable Utilization of Fujian Province, Fuzhou, Fujian China

**Keywords:** Amphioxus, Whole-genome resequencing, SNP, AlphaFold2

## Abstract

**Background:**

Amphioxus is a model organism for vertebrate evolutionary research. The significant contrast between morphological phenotypic similarity and high-level genetic polymorphism among amphioxus populations has aroused scientists' attention. Here we resequenced 21 amphioxus genomes to over 100X depth and mapped them to a haploid reference.

**Results:**

More than 11.5 million common SNPs were detected in the amphioxus population, which mainly affect genes enriched in ion transport, signal transduction and cell adhesion, while protein structure analysis via AlphaFold2 revealed that these SNPs fail to bring effective structural variants.

**Conclusions:**

Our work provides explanation for “amphioxus polymorphism paradox” in a micro view, and generates an enhanced genomic dataset for amphioxus research.

**Supplementary Information:**

The online version contains supplementary material available at 10.1186/s12863-022-01038-w.

## Background

The amphioxus, also known as lancelet, is the modern representative of the subphylum *Cephalochordata*, providing evolutionary insight into the origin of vertebrates [1, 2]. It is recognized that cephalochordates, urochordates, and vertebrates belonging to the phylum Chordata, evolved from a common ancestor that lived about 550 million years ago. Amphioxus has a vertebrate-like but simpler body plan, and different to most chordates, their genomes remained intact without any WGD events [2]. Hence, they are considered to be intermediate between invertebrates and vertebrates, and widely utilized as model organism for exploring vertebrate origin [1–6].

Previous studies have revealed a contradictory phenomenon that extreme phenotypic similarity and high-level genetic diversity [2, 3] [7–11] co-exist in amphioxus populations. Early publication claimed that the polymorphism rate of amphioxus might be as high as 5.37% [3], while they are commonly observed to share similar phenotypic characteristics [10], including body length, asymmetric body shape, number of oral cirri, etc. [12]. Taking *Branchiostoma belcheri* inhabiting Xiamen waters as an example, most adult individuals among these amphioxi present no apparent morphological differences, even females and males only differ in the reproductive organs [13], however genomic analysis revealed there might be up to 1 mutation site per 30 bases on their genome [10]. The “polymorphism paradox” has raised interests in studying the actual impact of amphioxus genomic mutation, which may provide not only evolutionary insights but also guidance for several potential applications, such as breeding [14].

Variant calling based on resequencing of main amphioxus species is an approach to seek the reasons behind “amphioxus polymorphism paradox”, for it helps researchers to get whole-genome variant distribution, where affected genes and regulatory elements can be identified for functional analysis. High-quality reference genome is the prerequisite of variant calling, while it is certain that representative amphioxus genomes have been successfully assembled [2, 3] [15, 16], some of which were assembled to chromosome level. In 2021, haploid genomes of three amphioxus species, *B. belcheri* (20 chromosomes), *B. japonicum* (18 chromosomes), and *B. floridae* (19 chromosomes), got completely resolved [16], further contributing to the amphioxus genomic toolbox. These works offer more options for subsequent resequencing experiments as researchers have higher chance to access the reference genome best representing their samples. On the basis above, several attempts have been made to build amphioxus variant datasets [10, 11], yet the low sequencing depth causing loss of polymorphisms [10] might make them difficult to provide reliable evidence.

In addition, the booming protein structure prediction algorithms have paved the way for exploring detailed variant effect on the protein level. As evidenced by the results of the biennial Critical Assessment of protein Structure Prediction (CASP), structure prediction has seen substantial progress in recent years [17]. In CASP14 (2020), the program AlphaFold2 [18] achieved a record score of 92.4, vastly more accurate than competing methods. Up to 2021, AlphaFold2 has predicted over 98.5% of human protein structures, and its results are recognized as reliable structure sources by some public protein databases such as UniProt. Compared to other prediction algorithms including RoseTTAFold [19], AlphaFold2 presents to be single in function and huge in performance overhead, while its unmatched accuracy maintains its “gold medal” in this field. As the rapid structure modelling solution challenging x-ray crystallography and cryo-electron microscopy, AlphaFold2 can directly transform sequences to structures, enabling researchers to observe SNP-brought structural difference intuitively.

Here we resequenced 21 amphioxus (*B. belcheri*) genomes to over 100X depth using the Illumina HiSeq X Ten platform, selected the new haploid genome [16] as the reference for joint SNP calling, and utilized AlphaFold2 to detect structural variant brought by SNPs. More than 11.5 million common SNPs were detected in the amphioxus population, which mainly affect genes enriched in ion transport, signal transduction and cell adhesion, while protein structure analysis via AlphaFold2 revealed that these SNPs fail to bring effective structural variants. Our work provides explanation for “amphioxus polymorphism paradox” in a micro view, and generates an enhanced genomic dataset for amphioxus research.

## Results

Reads passing quality control were mapped to the haploid reference genome assembled into 20 chromosomes using BWA [20], then GATK [21] was employed for SNP calling on each sample. The genomic mapping results are listed in Table 1. The offline read bases are 1,268.5G totally, and average sequencing depth is 124.66X. The polymorphism ratio is about 1 SNP per 30 bases, which does not differ much between individuals.

**Table 1:** The genomic mapping results of 21 *B. belcheri* individuals sequenced in this study

Sample (No.)	Sex	Bases (Gbp)	Mapped reads	Coverage (X)	Polymorphism
1	female	51.2	326,181,560 (95.54%)	105.98	3.33%
2	female	58.9	374,374,479 (95.38%)	121.75	3.29%
3	female	64.4	409,312,342 (95.31%)	132.87	3.27%
4	female	55.6	352,904,220 (95.22%)	114.52	3.30%
5	female	64.1	407,601,847 (95.35%)	132.58	3.29%
6	female	64.5	408,906,402 (95.11%)	132.69	3.28%
7	female	58.9	374,614,519 (95.36%)	121.59	3.30%
8	female	57.8	368,312,845 (95.5%)	119.58	3.33%
9	female	59.6	379,244,026 (95.42%)	123.22	3.30%
10	female	64.9	412,864,859 (95.42%)	134.34	3.30%
11	hermaphrodite	71.6	454,947,614 (95.26%)	147.84	3.25%
12	male	66.6	423,330,846 (95.35%)	137.78	3.26%
13	male	57.2	362,634,609 (95.18%)	117.91	3.29%
14	male	63.9	403,336,951 (94.65%)	131.02	3.29%
15	male	57	362,026,973 (95.28%)	117.72	3.31%
16	male	63.8	405,999,691 (95.5%)	131.86	3.29%
17	male	62.1	393,954,861 (95.12%)	128.32	3.28%
18	male	49.3	312,320,557 (95.12%)	101.62	3.32%
19	male	63	398,598,484 (94.92%)	129.28	3.28%
20	male	61.3	389,040,777 (95.27%)	126.09	3.29%
21	male	52.8	335,971,020 (95.41%)	109.25	3.35%

**Fig. 1:**
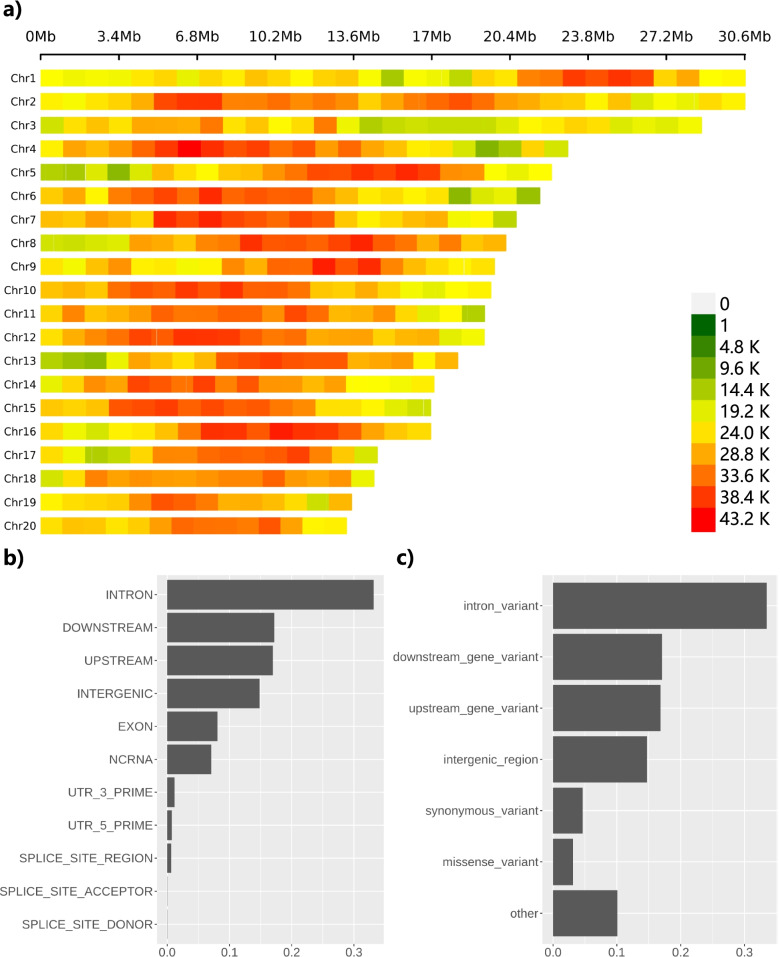
Distribution and effect of *B. belcheri* SNPs. **a** SNP distribution in *B. belcheri* chromosomes. Each portion of chromosome is 1 Mbp. **b** Statistics of SNPs detected in each type of genomic region. **c** Statistics of SNPs causing each variant type. Variant types are described by Sequence Ontology terms

Joint calling by GATK and PLINK [22] was launched after all individual SNP callings, where common SNPs (minor allele frequency > 0.05) were selected and got annotation from SnpEff [23]. There are totally 59,895,836 SNPs among 21 individuals, of which 11,541,148 are identified as common SNPs, approximately one per 34 bases in *B. belcheri* genome. Statistics of SNP distribution and effect are illustrated in Fig. 1. Figure 1a presents the SNP distribution centres on chromosomes in the form of heatmap. It’s obvious that for each chromosome there are one or two relatively wide distribution peaks. There is little difference among mutation rates of each chromosome, except for Chr1 and Chr3, whose polymorphism rates are relatively lower (Table S1).

Figure 1b-c reveal that most common SNPs are located in non-coding regions. 33.15% of them hit intron regions and 14.85% hit intergenic regions, and these two region types were found with most variants in previous studies [10]. Meanwhile it’s conspicuous that up to 34.2% get identified in up/downstream regions to genes, where cis-regulatory elements are located. SNPs hitting exon regions account for 8.07% (Fig. 1b), among which synonymous variants turn out to be slightly more than missense variants (Fig. 1c). Nonsynonymous variants having potential high impact on gene function are counted as a limited proportion of the total (Table S2). It should be mentioned that intron variants, synonymous variants as well as regulatory element variants mainly affect gene expression [24] rather than structure, thus numerous as they are, their impacts are considered not as high as nonsynonymous variants [23]. In addition, differences of SNP distributions among female, hermaphrodite and male amphioxus individuals are observed to be far from significant (Fig S1, Table S3), indicating that SNP distribution is not closely related to their sex.

**Fig. 2:**
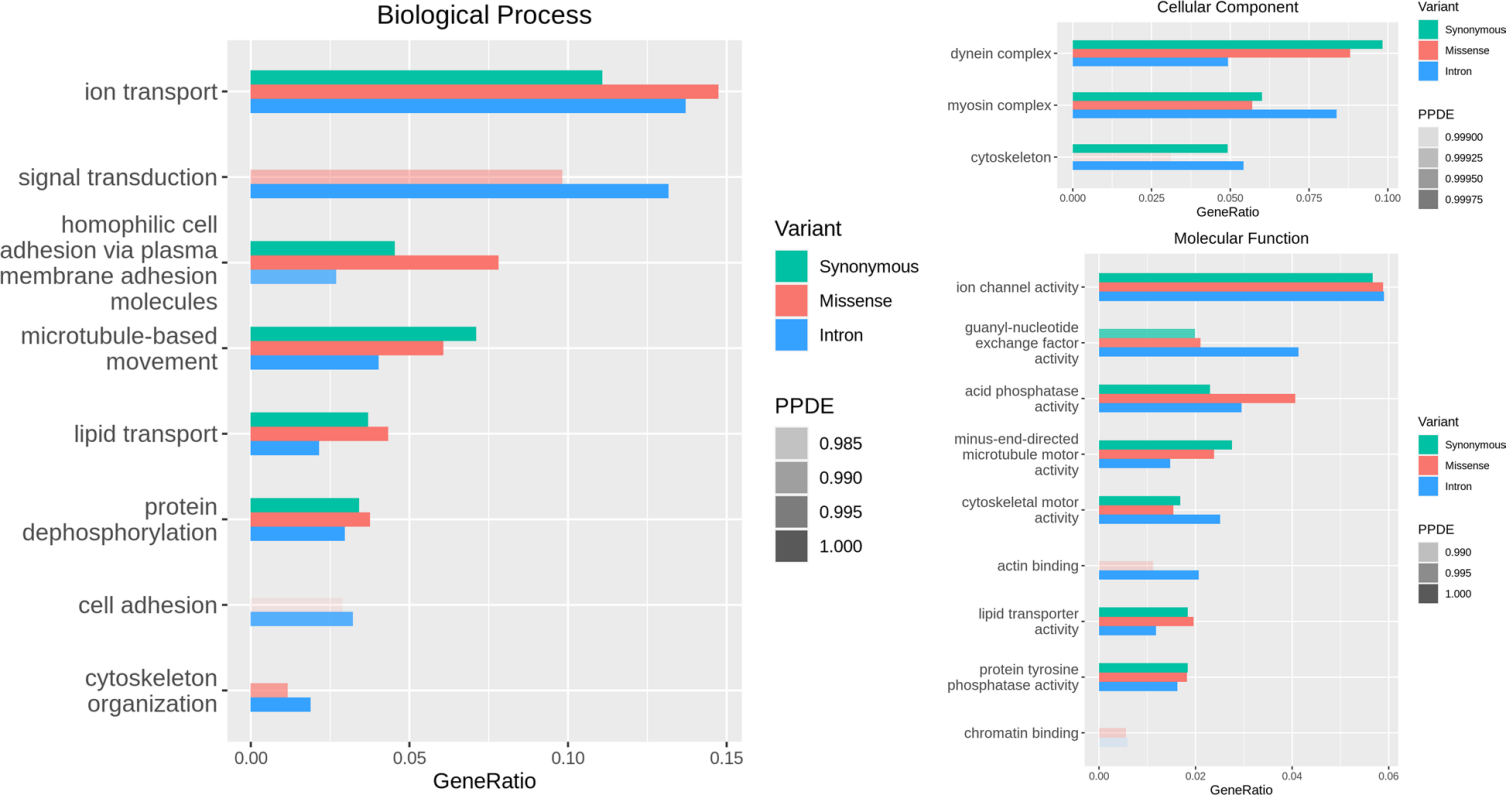
GO enrichment results of top-1000 genes with largest number of intron variants, synonymous variants, and missense variants. Enrichment plots are generated with categories marked by colour and statistical significance (PPDE = 1 – *P* value) marked by transparency. PPDE legend displays the gradient of transparency

GO enrichment analysis was separately performed on top-1000 genes with largest number of intron variants, synonymous variants and missense variants, results of which are summarized as Fig. 2. The top enriched terms of biological process and molecular function are related to ion transport, signal transduction and homophilic cell adhesion via plasma membrane adhesion molecules, where highly missense-mutated genes outnumber those synonymous-mutated. It indicates that variant selectivity might exist in related amphioxus gene families. Besides, enriched cellular component terms show that mutation frequently appears in amphioxus cytoskeleton along with its related complex. Enrichment results of these genes are generally consistent with their function distribution according to COG annotations (Fig S2).

**Fig. 3:**
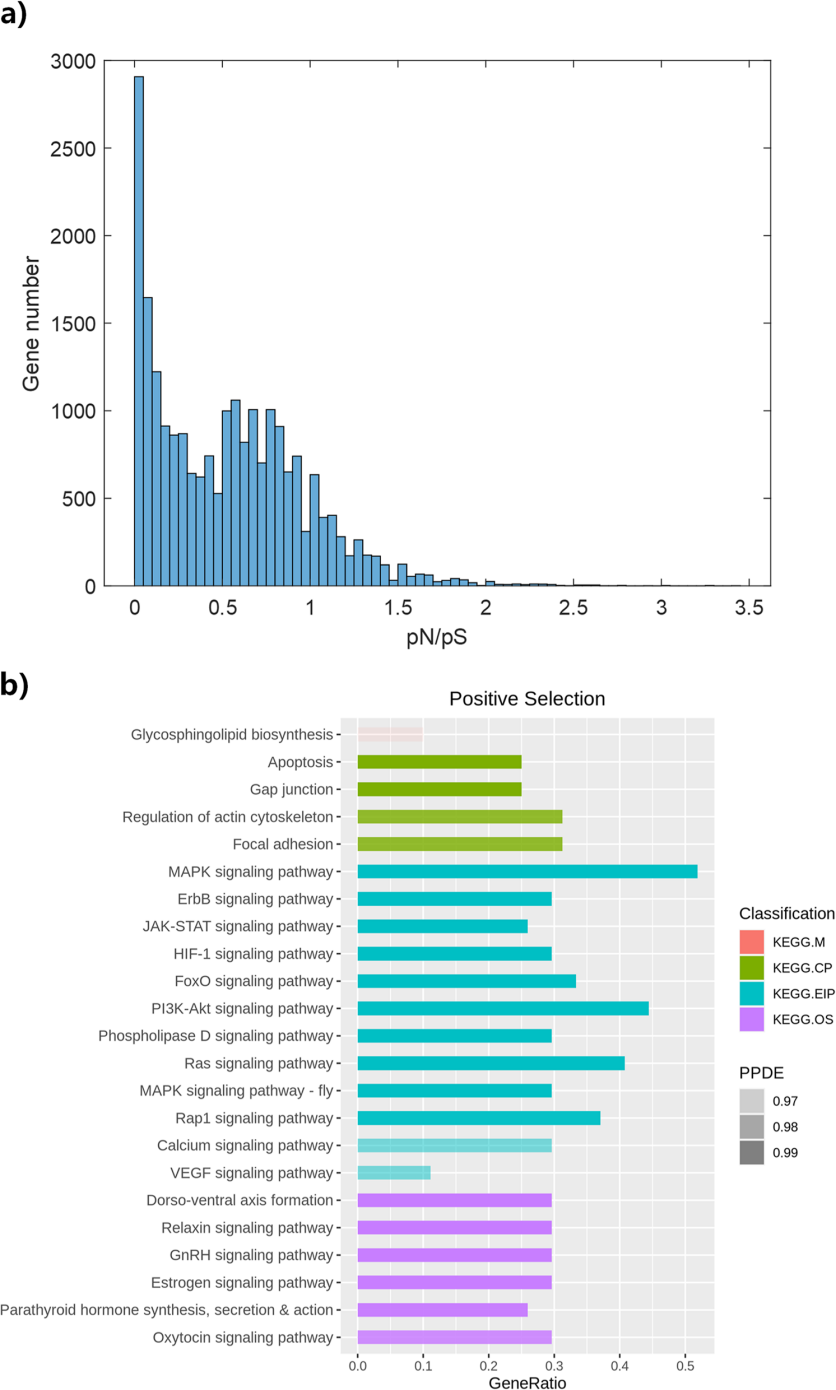
Results of pN/pS analysis. **a** Distribution of pN/pS ratios of genes with a minimum of five synonymous SNPs. **b** KEGG enrichment results of genes with pN/pS > 1.5. KEGG.M, KEGG.CP, KEGG.EIP, KEGG.OS are short for metabolism, cellular processes, environmental information processing and organismal systems in KEGG. Enrichment plots are generated with categories marked by colour and statistical significance (PPDE = 1 – *P* value) marked by transparency. PPDE legend displays the gradient of transparency

The pN/pS ratios of genes with a minimum of five synonymous SNPs were calculated, distribution of which is displayed in Fig. 3a. Genes with pN/pS > 1.5 account for about 2.14%, presenting to be a relatively large positive tail. KEGG enrichment analysis was performed on them, and results (Fig. 3b) reveal that these genes be involved in hormone regulation, mainly including growth-related pathways (MAPK, PI3K-Akt, etc.) and reproduction-related pathways (GnRH, Estrogen, Oxytocin, etc.). Meanwhile they might participate in response to hypoxic stress (HIF-1 and FoxO).

**Fig. 4:**
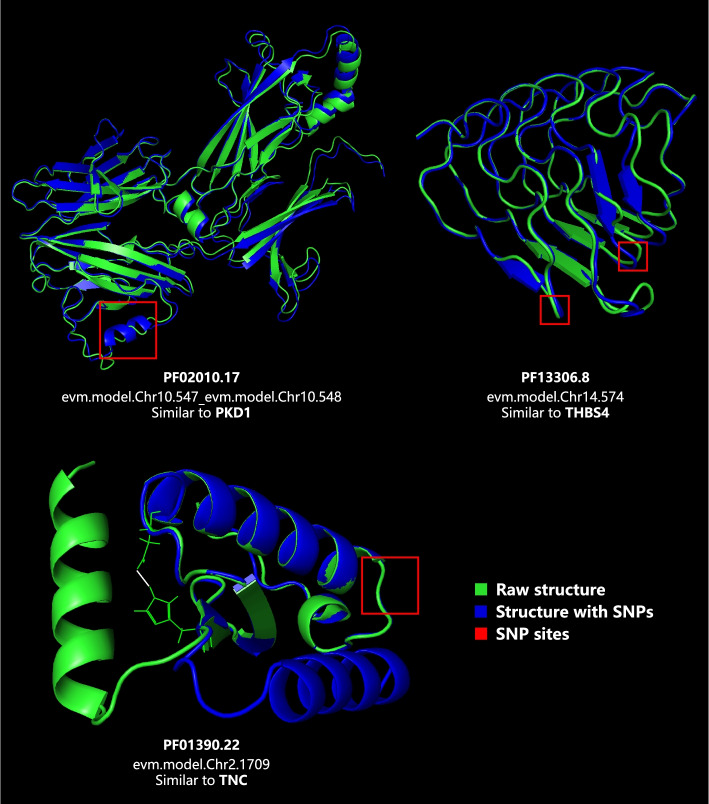
Examples of protein structure variation brought by SNPs. For each example, raw SNP-absent structure is marked by green and SNP-present structure is marked by blue. SNPs are located in the red-squared regions. Structures with SNPs are aligned to original structures for comparison, where overlapping green–blue regions can be recognized as unchanged

AlphaFold2 was employed to help evaluate protein structural variation brought by SNPs. Based on gene annotation, SNP distribution statistics and enrichment results, 100 domains were picked out as samples for structure analysis. Comparison between each raw-mutated structure pair reveals that only few SNPs could cause significant change in protein spatial structure, examples of which are displayed in Fig. 4. The largest variation is found in the SEA domain of a gene from Chr2, where SNPs at the beginning of the first α-helix interfere with the formation of a crucial hydrogen bond, triggering to conformational change. Several SNPs can also affect secondary structure such as α-helix and β-sheet, as observed in genes from Chr10 and Chr14. Except for them, other SNPs seem to modify intermolecular interaction at most, failing to exert great effects on protein structure, let alone the function (Data S2).

## Discussion

The polymorphism rate of *B. belcheri* in our results (about 3%) turns out to be larger than that of previous researches using low-depth sequencing data [10, 11], though still smaller than several reported high numbers (5.37% for *B. belcheri* and 4% for *B. floridae* of the same genus). The improvement of reference genome and sequencing method can exert positive influence on SNP calling, for it helps researchers to capture more mutation sites, thus reducing false negative rate and laying foundation for deeper studies on a larger sample size. In fact, genomic polymorphism rates of various marine lives are identified as high, for example, 1–1.5% in sea squirts (urochordate) [25], 4–5% in sea urchin (echinoderm) [26], 2–2.5% in oyster (lophotrochozoan) [27], etc. Generally, high mutation rate might offer them better natural physiological tolerance, enabling them to extend their species lifespan [28]. The actual impact of such numerous variants on these fantastic animals is a complex but valuable scientific question.

As for amphioxus, it indeed owns widely-distributed SNPs, while two factors limit their functional effect. For one thing, the majority of SNPs (more than 90%) hit non-coding regions, and those located in protein-coding regions mainly trigger to synonymous variants, in that pN/pS ratios of most genes (more than 75%) are below 1 (Fig S3), the symbol of neutral or purifying selection. This is consistent with other species for coding regions commonly maintain highly conserved to perform relevant functions and avoid potential damaging. For another, structure modelling reveals that most SNP-affected sequences are translated to proteins without significant structural changes. It seems that inside these living fossils, the role of translation is not only conversion, but also protection, in that effects of DNA-level mutation get weakened on the protein structure level to a certain extent. These above together shape amphioxus into phenotypically convergent species. Whether there is a similar situation in other marine organisms with high mutation rate needs to be further explored.

While it’s still discovered that in contrast to the overall variant distribution, genes identified as highly missense-mutated turn out to outnumber those synonymous-mutated in several pathways, mainly involved in signal transduction, ion transport and cell adhesion (See Table S4 for details). As the filter feeder, amphioxus utilize cilia to filter seawater and gather a variety of algae as food. They engulf food particles via phagocytic intracellular digestion mechanism [29, 30], which means they need to efficiently degrade algal toxin while intracellularly transforming algae into small molecules. Ion channels are the targets of many algal toxins [31], hence related genes inevitably suffer from high selective pressure, meanwhile their immune system and digestive system have to maintain active almost all the time. These may all contribute to the functional variation for survival. The SEA domain shown in Fig. 4, one of few samples detected with significant structural change in this work, has been recorded to participate in immune regulation and digestive enzyme production [32, 33], serving as evidence for the assumption above. Furthermore, pN/pS analysis sheds light on the positive selection acting in amphioxus endocrine-related genes, as illustrated in Fig. 3b. Previous studies indicate that amphioxus own several primitive regulatory axes, for example, their GH-IGF signalling system consisting of Hatschek’s pit and hepatic cecum regulates their growth and osmotic pressure [34, 35], analogous to pituitary-hepatic axis in vertebrates; brain vesicle, Hatschek’s pit and gonad constitute their reproductive endocrine axis, sharing functional similarity with HPG (hypothalamic-pituitary–gonadal) axis [36–38]; The coordination of their endostyle and Hatschek’s pit is potentially the early version of TSH-TH signalling system [39–41]; etc. Positive selection upon related genes might help to complicate and refine their signal transduction networks, so as to promote evolutionary processes in regulatory mechanisms.

In view of their extremely long history of existence and evolution, there might be few spaces for amphioxus to gain spontaneous genetic optimization now. From another perspective, it indicates the great threats from booming human activities, even the construction of a seawall could lead to the rapid disappearance of a hundreds-year-old *B. belcheri* fishing ground [42]. Therefore, the aim of studying amphioxus SNPs shouldn’t be confined to supplementing contents of biological treatise. Several variants hidden in our large dataset are probably the key to save amphioxus, of which scientists can take advantages to breed individuals and enlarge their population. Nevertheless, it’s bound to be an effort-consuming work to mine useful information from such seas of data, challenging the knowledge base and experimental infrastructure of any single group, besides an expanded sample size is required for subsequent confirmatory and translational researches.

## Conclusions

Our work not only sheds light on the distribution and effect of amphioxus SNPs but also makes progress in the explanation for “amphioxus polymorphism paradox”. It is proposed that influence of high-level genetic polymorphism be sharply weakened on the protein level. Numerous and selective as they are, amphioxus SNPs lack the macromolecular basis of impact on phenotypic characteristics.

It is expected that the upgraded sequencing technology with much deeper coverage, the reference genome with higher assembly level, and the advanced protein structure prediction algorithm make our new genomic dataset a valuable resource for exploring amphioxus biology as well as the origin of vertebrates. Due to limitation of computing power and storage, there remain lots of unmined information such as rare variants in it, thus joint efforts from further research are always welcome.

## Methods

### Additional annotation for reference genome

The haploid genome of *Branchiostoma belcheri* is provided by Fujian Key Laboratory of Special Marine Bio-resources Sustainable Utilization [16], including full sequences, protein-coding sequences, and annotation in GFF3 format (GCA_019207075.1, Data S1).

HMMER 3.1 package was employed for Pfam [43] annotation of reference genome. GO [44] annotations were converted from Pfam annotation via the external2go service at http://current.geneontology.org/ontology/external2go/pfam2go. KEGG and COG annotations were acquired from online services KAAS (https://www.genome.jp/kegg/kaas/) and eggNOG-mapper (http://eggnog-mapper.embl.de) [45–47].

### Sample preparation, DNA extraction, and sequencing

Twenty-one *B. belcheri* individuals, 10 male, 10 female and 1 hermaphrodite, were obtained from shoals (within 1km^2^) in Zhanjiang, Guangdong province during the summer breeding season, and kept at 28–32 °C at the Beihai Marine Station of Nanjing University, Guangxi, China.

Twenty-one samples were anesthetized and fixed by ethanol (100%, AR) before muscles were dissected. Genomic DNA was extracted separately using a QIAamp® DNA mini kit (Qiagen, Germany) following the standard manufacturer's protocol [29]. The purity and concentration of total DNA were determined with a NanoDrop spectrophotometer (NanoDrop, Wilmington, DE). DNA integrity was assessed by agarose gel electrophoresis. Briefly, the DNA sample was fragmented using a Covaris ultrasonic processor (Covaris, USA) to a size of ~ 350 bp, then the fragmented DNA was end repaired, “A”-tailed, and ligated with the full-length adaptor for Illumina sequencing with further PCR amplification. The concentrations of the constructed libraries were initially measured and diluted to 1 ng/μl by Qubit®2.0 (Life technologies, USA). Then, an Agilent Bioanalyzer 2100 system (Agilent, USA) was used to check the insert size of the libraries. To ensure the quality of these constructed libraries, the SYBR green qRT-PCR protocol was used with a Kapa Probe Fast qPCR kit (Kapa Biosystems, USA) to accurately dose the effective concentrations of the libraries. Finally, these libraries were sequenced on the Illumina HiSeq X Ten platform (Illumina, USA) by the Novogene Bioinformatics Institute, Beijing, China.

Quality control criteria [48] were applied to remove low-quality reads:

1. Removal of reads with more than 10% unidentified nucleotides (N);
2. Removal of reads containing more than 50% of bases with a Phred score ≤ 5;
3. Removal of putative PCR duplicate reads generated by PCR amplification.

Then sequences got further filtering via fastp [49] (*-q 20 -c*).

### Individual SNP calling

After removing low-quality reads, the clean paired-end reads were mapped to the haploid reference genome using BWA [20] (*-M -k 19*), then secondary or supplementary alignments were filtered by sambamba [50] (*- “ "not (secondary_alignment or supplementary”)" -p -l 9*).

The remaining mapped reads were sorted and converted into BAM format files using SAMtools [51]. PCR duplicates were marked using the GATK MarkDuplicates module.

Two rounds of individual SNP calling were performed using GATK, including following steps:

1. RealignerTargetCreator and IndelRealigner modules applied run to reduce the false-positive variants where alignment error occurred across overlapping reads;
2. HaplotypeCaller and VariantFiltration modules were applied to detect SNPs (QD < 10.0, MQ < 50.0, FS > 10.0, MQRankSum <  − 5.0, ReadPosRankSum <  − 8.0);
3. BaseRecalibrator and ApplyBQSR modules were applied to generate recalibrated bam files for each individual;
4. HaplotypeCaller module was applied again to detect variants (*–emit-ref-confidence GVCF*).

### Joint calling and SNP annotation

All GVCF files were processed by CombineGVCFs and GenotypeGVCFs modules to generate the population genotype file, then SNPs were selected using SelectVariants and VariantFiltration modules (QD < 2.0, MQ < 40.0, FS > 60.0, MQRankSum <  − 12.5, ReadPosRankSum <  − 8.0, QUAL < 30). Common SNPs in the population were selected by PLINK [22] (*–maf 0.05, –geno 0.05, –hwe 1e-4*), and got annotation by SnpEff [23]. In addition to annotation in VCF format, SnpEff generated the statistics of SNPs’ genomic locations and coding effect defined in SO [52] terms, which were extracted to plot SNP distribution heatmap and other statistic charts (Fig. 1, Fig S1).

### Gene functional enrichment analysis

GO enrichment analysis was performed on top-1000 (*P* value < 1e-5) genes with largest number of intron variant (SO:0,001,627), synonymous variant (SO:0,001,819), and missense variant (SO:0,001,583) using clusterProfiler [53] (*pvalueCutoff* = *0.05, pAdjustMethod* = *'BH', qvalueCutoff* = *0.2*).

The pN/pS ratios of genes were calculated referring to existing approach [54], and KEGG enrichment analysis using the same tool and parameters as GO analysis were performed on those with pN/pS > 1.5 and a minimum of five synonymous SNPs.

### Protein structural variation analysis

Sequences of missense-variant-enriched genes were extracted based on enrichment analysis and SNP statistics above, then 100 crucial protein domains according to Pfam annotation were chosen for structure analysis. For each raw-mutated sequence pair, AlphaFold2 [18] (*default parameters*) was run to get the highest-score structures (*ranked_0.pdb*) to make comparison.

## Supplementary Information

**Additional file 1: Data S1.** Protein-coding sequences and annotation of the haploid reference genome. **Data S2.** Samples for AlphaFold2 analysis. **Fig S1.** Pie charts of SNPs detected in each type of genomic region in amphioxus of different sexes. **Fig S2.** Statistics of COG functional classes of top-1000 genes with largest number of synonymous variants (a), missense variants (b), and intron variants (c). A: RNA processing and modification; B: Chromatin structure and dynamics; C: Energy production and conversion; D: Cell cycle control, cell division, chromosome partitioning; E: Amino acid transport and metabolism; F: Nucleotide transport and metabolism; G: Carbohydrate transport and metabolism; H: Coenzyme transport and metabolism; I: Lipid transport and metabolism; J: Translation, ribosomal structure and biogenesis; K: Transcription; L: Replication, recombination and repair; M: Cell wall/membrane/envelope biogenesis; N: Cell motility; O: Posttranslational modification, protein turnover, chaperones; P: Inorganic ion transport and metabolism; Q: Secondary metabolites biosynthesis, transport and catabolism; R: General function prediction only; S: Function unknown; T: Signal transduction mechanisms; U: Intracellular trafficking, secretion, and vesicular transport; V: Defense mechanisms; W: Extracellular structures; X: Unnamed protein; Y: Nuclear structure; Z: Cytoskeleton. **Fig S3.** Box plot of total pN/pS ratio distribution. **Table S1.** Number of common SNPs on each chromosome. **Table S2.** Number of variants affecting each gene calculated via SnpEff. **Table S3.** Statistics of SNPs detected in each type of genomic region in each amphioxus sample. **Table S4.** Full tables of GO enrichment results.

## Data Availability

Raw data from our whole-genome sequencing are available at NCBI (PRJNA742127). Total SNP set is available at figshare (https://doi.org/10.6084/m9.figshare.18833234.v1).

## Abbreviations

WGD: Whole-Genome Duplication
SNP: Single Nucleotide Polymorphism
GO: Gene Ontology
SO: Sequence Ontology
